# High prevalence of hypertension among smokers of conventional and e-cigarette: Using the nationally representative community dwelling survey

**DOI:** 10.3389/fpubh.2022.919585

**Published:** 2022-10-17

**Authors:** Soo Young Kim, Sung Hoon Jeong, Hye Jin Joo, Minah Park, Eun-Cheol Park, Jung Hyun Kim, Junbok Lee, Jaeyong Shin

**Affiliations:** ^1^Department of Public Health, Yonsei University, Seoul, South Korea; ^2^Institute of Health Services Research, Yonsei University, Seoul, South Korea; ^3^Department of Preventive Medicine, Yonsei University College of Medicine, Seoul, South Korea; ^4^Health IT Center, Yonsei University Health System, Seoul, South Korea

**Keywords:** exclusive e-cigarette use, hypertension, dual smoking, cigarette type, e-cigarette

## Abstract

This study aimed to clarify the association between hypertension and conventional cigarette and electronic cigarette (e-cigarette) use, together or individually. A total of 275,762 participants were included, of which 120,766 were men and 154,996 were women. The data were drawn from the Korea Community Health Survey conducted in 2019. A multiple logistic regression model was used to examine the association between hypertension and types of smoking. Hypertension was defined as systolic blood pressure higher than 140 mmHg or diastolic blood pressure higher than 90 mmHg. Based on the types of smoking, participants were grouped as dual smokers of conventional and e-cigarettes, e-cigarette only smokers, conventional cigarette only smokers, past-smokers, and non-smokers. Compared to non-smokers, dual smokers presented the highest odds ratio for hypertension in the male [odds ratio (OR): 1.24, confidence interval (CI): 1.10 to 1.39] and female groups (OR: 1.44 CI: 0.96 to 2.15). According to the Cochran-Mantel-Haenszel test, the two-sided *p*-value of < 0.001 indicated an overall statistically significant association between types of smoking and hypertension. Use of both cigarette types was statistically significant in the male group, but only the use of conventional cigarettes and past smoking were statistically significant in the female group. Among smokers of the two cigarette types, those who were dual smokers of e-cigarettes and conventional cigarettes were the most likely to have the highest prevalence of hypertension.

## Introduction

Electronic cigarette (e-cigarette) use has been spreading worldwide in recent years. An e-cigarette is a reusable cigarette, charging nicotine and other cigarette components in the form of a liquid. Since e-cigarettes do not contain the scent of conventional cigarettes, they are commonly favored by adolescents, resulting in an increased prevalence of smoking among the youth. Therefore, e-cigarettes should be thoroughly examined before being recommended as an alternative when helping people to quit smoking safely.

Compared to conventional cigarettes, there are fewer studies about e-cigarettes that assess a large enough sample size to ensure generalizability. E-cigarette devices create aerosols, which many people call “vaper.” This usually contains glycerin, propylene glycol, flavorings, and nicotine (1). The liquid components can vary; for instance, one of the flavoring components contains diacetyl, which is known to cause bronchiolitis obliterans, an irreversible respiratory disease (2, 3). Additionally, e-cigarettes can be harmful mentally as well as physically. A study showed that those who only used e-cigarettes were more likely to have suicidal ideation and planning than dual smokers (4). In another study, e-cigarette use was associated with adverse mental health status, particularly in women (5). As seen in previous studies, there are plural research showing the harmful effects of e-cigarettes. Therefore, further investigation on the safety of e-cigarettes is needed.

In this paper, association between cigarette use and hypertension will be investigated. Hypertension is one of the most well-known chronic disease. Burden of chronic disease has many layers of problems. Firstly, as one of the previous studies indicate, advances in research and health care have reduced mortality from acute diseases and extended life expectancy. Nevertheless, the layers of emotional stress that chronic disease give became a type of problem to cope with for the patients (6). Secondly, the rising cost of medical care is highly dependent on chronic disease patients. The indirect cost is to rise because, as the proportion of patients with chronic health conditions result in limited economic activity, eventually holding back the economic prosperity (7). On this account, we could conclude that there is great harm of cigarettes, and especially in e-cigarettes that could promote one to suffer from hypertension and other chronic diseases.

Smoking and hypertension have a well-known causal relationship (8–10). However, there are two contrary claims regarding e-cigarette smoking (11, 12). One study showed that blood pressure (BP) was controlled in smokers with arterial hypertension who switched to electronic cigarettes (11). In this study, e-cigarettes were used as a way to quit conventional smoking. This showed a considerable reduction in conventional cigarette use in e-cigarette users. Compared to regular conventional cigarette smokers, e-cigarette smokers experienced a reduction in median systolic BP (SBP) from 140 to 130 mmHg (*p* < 0.001) and diastolic BP (DBP) from 86 to 80 mmHg (*p* = 0.006). The study concluded that the decline in cigarette consumption was associated with a significant reduction in systolic and diastolic BP compared to the baseline at 12 months (11). However, another cross sectional study concluded that current users of e-cigarettes had higher odds for the prevalence of hypertension [odds ratio (OR) = 1.31 95% confidence interval (CI) (1.05–1.63)] than current conventional cigarette users [OR = 1.27 95% CI (1.10–1.47)]. Respondents who were concurrently smoking both conventional cigarettes and e-cigarettes had the highest odds of having hypertension [OR = 1.77 95% CI (1.32–2.39)], compared to non-smokers (12).

Using the data of a substantial sample size from the Korean Community Health Service (KCHS), which could represent the whole Korean population, we studied the association between e-cigarette use and hypertension according to different smoking practices.

## Materials and methods

### Data

Data used in the study were accessed from the KCHS implemented in 2019 by the Korea Center for Disease Control and Prevention Agency (KDCA) (13). The KCHS is a nationwide survey acquired annually to evaluate health behavior and health status since 2008 (14). The data from these surveys are used to monitor and estimate the prevalence of chronic diseases in the South Korean population (15, 16).

### Participants

In total, 275,840 individuals over the age of 19 years were included in this study. Those aged below 18 years were excluded since this study aimed to investigate the prevalence of hypertension in adults. Moreover, only the participants who did not take hypertension-related medication were included. The KCHS received the Korea Centers for Disease Control and Prevention (KCDC) IRB approval (2016-10-01-P-A) in 2016. From 2017, the ethics approval for the KCHS was waived by the KCDC IRB as it does not fall under human subject research based on the enforcement rule of the bioethics and safety act.

### Variables

The dependent variable was the prevalence of hypertension, determined by the SBP cutoff point of 140 mmHg or DBP of 90 mmHg (17). BP was acquired through the measurement section of data collection. The SBP and DBP were each measured three times, and the average was then calculated.

The main independent variable of interest was the type of cigarette used. We divided the variable of interest into five groups: current dual smokers of conventional and e-cigarettes were included in group 1; e-cigarette only smokers, group 2; current conventional cigarette only smokers, group 3; past smokers of either conventional or e-cigarette, group 4; and non-smokers, group 5.

To define the smokers and past smokers smoking, we used the questions asked in the KCHS. Those who answered yes to “Have you ever smoked certain type of cigarette?” and yes to “Are you currently smoking certain type of cigarette?” were defined as current smokers. If the respondent answered yes to the prior question and no to latter question, they were defined as past smokers. Lastly, those who responded no to either of the questions were set as those who never smoked. For past smokers, abstinence time requirement for past smokers was not recorded in the survey.

We controlled for covariates such as demographic and socioeconomic factors, health behaviors, and health conditions of the participants. The demographic factors were age (19–60, at 10-year intervals) and sex. The socioeconomic factors included education level (below middle school, high school, and university or higher), region (urban and rural areas), marital status (living with or without a spouse), occupation (white, pink, blue-collar, and unemployed), and household income in quartiles. Health behaviors included drinking habits (yes or no) and performing physical activity. Health conditions included the perceived amount of stress (a lot or less) and body mass index (underweight/normal and overweight/obese). Since this study examined smoking, we also included the age of onset of conventional smoking and calculated the pack-year of conventional smokers.

### Statistical analysis

The frequency of use of the two cigarette types and hypertension stratified by covariates was compared using the chi-squared test. Multiple logistic regression analysis was carried out to examine the association between the type of smoking practice and hypertension.

The data were analyzed and further stratified by sex. The sex difference in smoking was considerable. Therefore, we believed that it would be more precise to divide participants into two groups based on sex (18). The predisposition to smoking was not only seen in Asian countries but also in European countries (19, 20).

The results are reported using ORs and CIs. A *p*-value < 0.05 was considered statistically significant. The SAS 9.4 (SAS Institute Inc; Cary, North Caroline) software was used for each part of the statistical analysis.

## Results

According to the results of our study, 18.3% of the participants were smokers. However, when comparing this rate to other national statistics [20.2% in the Korea National Health and Nutrition Examination Survey (KNHANES) and 21.5% in the national statistics webpage managed by the government], our study results are slightly lower (21). Moreover, the proportion who smoked was 16.3% men and 2% in women. The rate from official national sources was two-fold higher than that of our study [KNHANES: men, 34.7%; women, 5.9%; Korea Statistics (KOSTAT): men, 35.7%; women, 6.7%].

Table 1 shows the general characteristics of the study participants. From the total of 275,762 participants, 120,766 were men and 154,996 were women. The proportion of participants with hypertension was 13.6% for men and 9.4% for women.

**Table 1:** General characteristics of the study population.

**Variables**	**Hypertension** ^**b**^											
	**Male**						**Female**					
	**Total**	**Yes**		**No**		***P-*value**	**Total**	**Yes**		**No**		***P-*value**
	***N***	***N***	**%**	***N***	**%**		***N***	***N***	**%**	***N***	**%**	
Total (*N* = 275,762)	120,766	16,371	13.6	104,395	86.4		154,996	14,640	9.4	140,356	90.6	
Types of cigarette use						< 0.001						< 0.001
Dual smoker	2,880	382	13.3	2,498	86.7		388	26	6.7	362	93.3	
E-cigarette only	1,048	134	12.8	914	87.2		144	10	6.9	134	93.1	
Conventional only	41,024	6,071	14.8	34,953	85.2		4,864	590	12.1	4,274	87.9	
Past smoker	43,798	6,260	14.3	37,538	85.7		4,844	496	10.2	4,348	89.8	
Non-smoker	32,016	3,524	11.0	28,492	89.0		144,756	13,518	9.3	131,238	90.7	
Age (years)						< 0.001						< 0.001
19–29	16,206	1,119	6.9	15,087	93.1		19,816	565	2.9	19,251	97.1	
30–39	18,704	2,212	11.8	16,492	88.2		22,576	1,131	5.0	21,445	95.0	
40–49	23,298	3,449	14.8	19,849	85.2		29,694	2,270	7.6	27,424	92.4	
50–59	23,700	3,633	15.3	20,067	84.7		33,180	3,405	10.3	29,775	89.7	
≥60	38,858	5,958	15.3	32,900	84.7		49,730	7,269	14.6	42,461	85.4	
Marital status						< 0.001						0.7251
Living with spouse	82,556	11,420	13.8	71,136	86.2		100,318	9,463	9.4	90,855	90.6	
Living without spouse	38,210	4,951	13.0	33,259	87.0		54,678	5,177	9.5	49,501	90.5	
Region						< 0.001						< 0.001
Metropolitan city	36,592	4,723	12.9	31,869	87.1		50,010	4,069	8.1	45,941	91.9	
Rural	84,174	11,648	13.8	72,526	86.2		104,986	10,571	10.1	94,415	89.9	
Occupational categories^a^						< 0.001						< 0.001
White	29,380	3,645	12.4	25,735	87.6		32,588	1,858	5.7	30,730	94.3	
Pink	13,618	1,752	12.9	11,866	87.1		27,156	2,456	9.0	24,700	91.0	
Blue	49,330	7,190	14.6	42,140	85.4		28,596	3,500	12.2	25,096	87.8	
Inoccupation	28,438	3,784	13.3	24,654	86.7		66,656	6,826	10.2	59,830	89.8	
Educational level						< 0.001						< 0.001
Middle school or less	26,230	4,092	15.6	22,138	84.4		47,602	7,083	14.9	40,519	85.1	
High school	37,784	5,625	14.9	32,159	85.1		46,182	4,276	9.3	41,906	90.7	
College or over	56,752	6,654	11.7	50,098	88.3		61,212	3,281	5.4	57,931	94.6	
Household income						< 0.001						< 0.001
Low	27,102	4,163	15.4	22,939	84.6		39,128	5,390	13.8	33,738	86.2	
Mid-low	21,040	3,058	14.5	17,982	85.5		25,956	2,651	10.2	23,305	89.8	
Mid-high	34,386	4,626	13.5	29,760	86.5		41,216	3,360	8.2	37,856	91.8	
High	38,238	4,524	11.8	33,714	88.2		48,696	3,239	6.7	45,457	93.3	
Obesity status (BMI)						< 0.001						< 0.001
Normal	114,292	15,045	13.2	99,247	86.8		149,402	13,699	9.2	135,703	90.8	
Overweight and obese	6,474	1,326	20.5	5,148	79.5		5,594	941	16.8	4,653	83.2	
Physical activity						< 0.001						< 0.001
Yes	16,371	608	3.7	15,763	96.3		5,032	414	8.2	4,618	91.8	
No	104,395	4,420	4.2	99,975	95.8		149,964	14,226	9.5	135,738	90.5	
Alcohol use						< 0.001						< 0.001
Yes	92,692	12,836	13.8	79,856	86.2		59,842	6,737	11.3	53,105	88.7	
No	28,074	3,535	12.6	24,539	87.4		95,154	7,903	8.3	87,251	91.7	
Perceived stress						< 0.001						< 0.001
A lot	26,092	3,498	13.4	22,594	86.6		37,180	3,282	8.8	33,898	91.2	
Less	94,674	12,873	13.6	81,801	86.4		117,816	11,358	9.6	106,458	90.4	

^a^Three groups (white, pink, blue) based on the International Standard Classification Occupations codes. Inoccupation group includes housewives. ^b^(systolic pressure ≥ 140 mmHg or diastolic pressure ≥ 90 mmHg).

Table 2 presents the results of logistic regression. Men who smoked e- and conventional cigarettes simultaneously (group 1) and those who smoked only e-cigarettes (group 2) had the highest ORs associated with hypertension [group 1 OR = 1.24, 95% CI (1.11–1.39), group 2 OR = 1.22, 95% CI (1.02–1.48)]. Women with dual smoking (group 1) had the highest OR [OR = 1.44, 95% CI (0.96–2.16)], followed by women who were only e-cigarette smokers [group 2; OR = 1.41, 95% CI (0.74–2.70)]. Although the OR was the maximum, women with dual smoking and e-cigarette only smokers were statistically insignificant. The conventional cigarette only group (group 3) and former smoking group (group 4) were the next to follow in the sex-stratified statistics. These were the only statistically significant groups [group 3 OR = 1.35, 95% CI (1.24–1.48), group 4 OR = 1.11, 95% CI (1.11–1.35)].

**Table 2:** Results of factors associated with hypertension by smoking type.

**Variables**	**Hypertension** ^**b**^			
	**Male**		**Female**	
	**OR**	**95% CI**	**OR**	**95% CI**
**Total**
**Types of cigarette use**
Dual smoker	1.24	1.11–1.39	1.44	0.96–2.16
E-cigarette only	1.22	1.02–1.48	1.41	0.74–2.70
Conventional only	1.16	1.11–1.22	1.35	1.24–1.48
Past smoker	1.09	1.04–1.14	1.23	1.11–1.35
Non smoker	1.00		1.00	
**Age (years)**
19–29	1.00		1.00	
30–39	2.01	1.86–2.18	1.85	1.66–2.06
40–49	2.73	2.52–2.95	2.82	2.55–3.11
50–59	2.94	2.71–3.19	3.48	3.15–3.85
≥60	3.01	2.77–3.28	4.23	3.81–4.70
**Marital status**
Living with spouse	1.00		1.00	
Living without spouse	1.27	1.22–1.33	1.07	1.03–1.12
**Region**
Metropolitan city	1.00		1.00	
Rural	1.03	0.99–1.07	1.10	1.06–1.14
**Household income**
Low	1.15	1.09–1.22	1.21	1.14–1.28
Mid-low	1.14	1.08–1.20	1.13	1.06–1.19
Mid-high	1.09	1.05–1.14	1.07	1.02–1.13
High	1.00		1.00	
**Educational level**
Middle school or less	1.08	1.01–1.14	1.47	1.37–1.57
High school	1.10	1.05–1.15	1.22	1.16–1.29
College or over	1.00		1.00	
**Occupational categories** ^**a**^
White	1.00		1.00	
Pink	0.99	0.93–1.06	1.05	0.98–1.12
Blue	1.01	0.96–1.06	1.06	0.99–1.14
Inoccupation	1.03	0.97–1.09	1.06	1.00–1.13
**Alcohol use**
Yes	1.27	1.22–1.33	1.05	1.02–1.10
No	1.00		1.00	
**Physical activity**
Yes	1.00		1.00	
No	1.08	0.99–1.18	1.09	0.98–1.21
**Perceived stress**
A lot	1.00	0.95–1.04	1.01	0.97–1.05
Less	1.00		1.00	
**Obesity status (BMI)**
Normal or underweight	1.00		1.00	
Overweight and obese	2.02	1.89–2.15	1.99	1.85–2.15

^a^Three groups (white, pink, blue) based on the International Standard Classification Occupations codes. Inoccupation group includes housewives. ^b^(systolic pressure ≥ 140 mmHg or diastolic pressure ≥ 90 mmHg).

Table 3 shows the results of subgroup analysis stratified by independent variables. Among all the covariates that were modified, only the sociodemographic variables are presented in the table. Overall, the highest odds were found in dual or e-cigarette only smoking groups (group 1 and 2) in each variable for men. Women showed the same tendency as men. However, a few variables such as age group (30 and 40s) showed the highest odds in the conventional only cigarette smokers (group 3) and those who lived with a spouse or were unemployed. In both men and women, those who had educational level of college or over were more likely to have hypertension in those who smoke e-cigarette only [men OR: 1.32, 95% CI (1.05–1.65); female OR: 3.34, 95% CI (1.58–7.06)]. Moreover, among all the other covariates, all household income level particularly had dispersed tendency of having the highest OR across all types of cigarette use in both men and women.

**Table 3:** The results of subgroup analysis stratified by independent variables.

**Variables**	**Hypertension** ^**b**^								
	**Types of cigarette use**								
	**Dual**		**E-cigarette only**		**Conventional only**		**Past smokers**		**Non-smoker**
	**OR**	**95% CI**	**OR**	**95% CI**	**OR**	**95% CI**	**OR**	**95% CI**	**OR**
**MALE**									
**Age (years)**
19–29	1.40	(1.09–1.79)	0.99	(0.59–1.64)	1.27	(1.09–1.47)	1.15	(0.93–1.42)	1.00
30–39	1.24	(0.99–1.54)	1.36	(1.01–1.83)	1.27	(1.13–1.42)	1.12	(0.98–1.27)	1.00
40–49	1.11	(0.89–1.38)	1.03	(0.73–1.46)	1.07	(0.96–1.19)	1.12	(1.01–1.25)	1.00
50–59	1.32	(0.96–1.81)	1.78	(1.01–3.11)	1.10	(0.99–1.22)	1.10	(0.99–1.22)	1.00
≥60	1.35	(0.83–2.18)	1.61	(0.65–4.00)	1.14	(1.04–1.24)	1.02	(0.95–1.10)	1.00
**Marital status**
Living with spouse	1.21	(1.03–1.42)	1.21	(0.96–1.53)	1.12	(1.06–1.19)	1.06	(1.01–1.12)	1.00
Living without spouse	1.27	(1.08–1.51)	1.22	(0.90–1.67)	1.22	(1.13–1.32)	1.15	(1.05–1.26)	1.00
**Region**
Metropolitan city	1.36	(1.12–1.64)	1.33	(0.98–1.79)	1.17	(1.08–1.28)	1.07	(0.98–1.16)	1.00
Rural	1.18	(1.02–1.36)	1.17	(0.92–1.49)	1.15	(1.09–1.22)	1.10	(1.04–1.16)	1.00
**Household income**
Low	1.24	(0.88–1.75)	1.18	(0.62–2.26)	1.13	(1.03–1.24)	0.98	(0.90–1.08)	1.00
Mid-low	1.13	(0.84–1.53)	1.04	(0.60–1.81)	1.19	(1.07–1.33)	1.13	(1.01–1.26)	1.00
Mid-high	1.20	(0.98–1.47)	1.11	(0.80–1.55)	1.12	(1.03–1.22)	1.09	(0.99–1.19)	1.00
High	1.34	(1.12–1.60)	1.40	(1.06–1.84)	1.19	(1.09–1.30)	1.17	(1.07–1.27)	1.00
**Educational level**
Middle school or less	1.23	(0.73–2.05)	1.07	(0.31–3.70)	1.14	(1.03–1.27)	1.05	(0.95–1.15)	1.00
High school	1.13	(0.92–1.38)	1.06	(0.74–1.52)	1.05	(0.97–1.14)	1.02	(0.93–1.11)	1.00
College or over	1.28	(1.11–1.49)	1.32	(1.05–1.65)	1.22	(1.14–1.30)	1.16	(1.08–1.24)	1.00
**Occupational categories** ^**a**^
White	1.25	(1.02–1.53)	1.25	(0.94–1.67)	1.17	(1.07–1.29)	1.15	(1.05–1.26)	1.00
Pink	1.39	(1.06–1.82)	1.40	(0.91–2.14)	1.21	(1.05–1.40)	1.10	(0.94–1.28)	1.00
Blue	1.16	(0.95–1.40)	1.04	(0.73–1.50)	1.09	(1.02–1.18)	1.08	(1.00–1.16)	1.00
Unemployed	1.24	(0.88–1.76)	1.33	(0.75–2.35)	1.22	(1.10–1.34)	1.03	(0.93–1.13)	1.00
**FEMALE**									
**Age (years)**
19–29	1.52	(0.79–2.90)	2.57	(1.01–6.55)	1.48	(1.03–2.12)	1.19	(0.79–1.80)	1.00
30–39	1.01	(0.31–3.30)	1.27	(0.38–4.19)	1.60	(1.22–2.10)	1.12	(0.88–1.44)	1.00
40–49	1.29	(0.58–2.84)	0.80	(0.19–3.38)	1.37	(1.13–1.67)	1.23	(0.99–1.53)	1.00
50–59	2.24	(0.84–5.95)	< 0.001	< 0.001–>999.999	1.41	(1.19–1.68)	1.35	(1.09–1.68)	1.00
≥60	5.41	(0.34–86.60)			1.17	(1.00–1.37)	1.19	(1.02–1.39)	1.00
**Marital status**
Living with spouse	0.91	(0.42–1.97)	0.25	(0.03–1.81)	1.30	(1.13–1.49)	1.28	(1.12–1.46)	1.00
Living without spouse	1.88	(1.17–3.03)	2.87	(1.42–5.80)	1.44	(1.27–1.62)	1.17	(1.02–1.35)	1.00
**Region**
Metropolitan city	1.34	(0.68–2.65)	2.30	(0.91–5.82)	1.55	(1.32–1.82)	1.28	(1.07–1.52)	1.00
Rural	1.51	(0.91–2.49)	1.03	(0.41–2.56)	1.27	(1.13–1.41)	1.21	(1.08–1.36)	1.00
**Household income**
Low	1.73	(0.78–3.83)	4.20	(1.18–14.98)	1.30	(1.13–1.50)	1.18	(1.00–1.38)	1.00
Mid-low	1.29	(0.52–3.23)	0.69	(0.09–5.15)	1.38	(1.13–1.67)	1.21	(0.96–1.52)	1.00
Mid-high	0.77	(0.28–2.12)	1.00	(0.24–4.20)	1.33	(1.10–1.60)	1.35	(1.12–1.63)	1.00
High	1.95	(1.01–3.78)	1.31	(0.47–3.67)	1.49	(1.18–1.88)	1.16	(0.93–1.46)	1.00
**Educational level**
Middle school or less	1.87	(0.54–6.54)	< 0.001	< 0.001–>999.999	1.25	(1.08–1.45)	1.20	(1.03–1.40)	1.00
High school	1.51	(0.86–2.64)	0.45	(0.11–1.84)	1.37	(1.19–1.57)	1.24	(1.06–1.45)	1.00
College or over	1.15	(0.58–2.28)	3.34	(1.58–7.06)	1.45	(1.15–1.83)	1.19	(0.98–1.45)	1.00
**Occupational categories** ^**a**^
White	0.65	(0.20–2.09)	2.45	(0.74–8.19)	1.50	(1.13–2.01)	1.35	(1.05–1.75)	1.00
Pink	2.28	(1.29–4.03)	1.65	(0.64–4.23)	1.47	(1.24–1.76)	1.22	(0.97–1.54)	1.00
Blue	1.79	(0.62–5.13)	< 0.001	< 0.001–>999.999	1.26	(1.03–1.55)	1.25	(1.00–1.57)	1.00
Unemployed	0.93	(0.38–2.31)	0.84	(0.20–3.50)	1.30	(1.13–1.49)	1.18	(1.03–1.36)	1.00

^a^Three groups (white, pink, blue) based on the International Standard Classification Occupations codes. Unemployed group includes housewives. ^b^(systolic pressure ≥ 140 mmHg or diastolic pressure ≥ 90 mmHg).

The logistic regression method indicated that the types of smoking and hypertension were related. Consistent with this, the Cochran-Mantel-Haenszel test for trend analysis also revealed that there is potential trend between types of smoking and hypertension. The two-sided *p*-value of < 0.001 showed there is an overall statistically significant association between the variable of interest (smoking) and the dependent variable (hypertension).

Table 4 presents the analysis of variables of interest stratified by pack-year and age of onset. In the male group, each group was statistically significant and more likely to experience hypertension than those who had not smoked, except for the e-cigarette only group (group 2). In group 2, the statistics were as follows: [pack-year < 20, OR = 1.16, 95% CI (0.92–1.45); 20 ≤ pack-year < 30, OR = 1.40, 95% CI (0.88–2.23); and pack-year > 30, OR = 1.72, 95% CI (0.99–3.00)]. Pack-year was calculated for group 2 and other groups, as past conventional cigarette smokers were included in the group.

**Table 4:** Subgroup analysis by pack year.

**Variables**	**Hypertension** ^**a**^			
	**Male**		**Female**	
	**OR**	**95% CI**	**OR**	**95% CI**
**Pack year**
**Pack year** **<** **20**
Dual smoker	1.21	1.05–1.40	1.47	0.92–2.33
E–cigarette only	1.16	0.92–1.45	1.40	0.64–3.04
Conventional only	1.13	1.07–1.19	1.41	1.27–1.58
Past smoker	1.06	1.01–1.12	1.22	1.10–1.35
**20** **≤Pack year** **<** **30**
Dual smoker	1.37	1.06–1.78	0.82	0.11–6.37
E-cigarette only	1.40	0.88–2.23	< 0.001	< 0.001–>999.999
Conventional only	1.20	1.12–1.29	1.36	1.03–1.80
Past smoker	1.14	1.05–1.23	1.34	0.89–2.01
**Pack year** **>** **30**
Dual smoker	1.18	0.93–1.50	1.54	0.61–3.87
E-cigarette only	1.72	0.99–3.00	< 0.001	< 0.001–>999.999
Conventional only	1.18	1.11–1.25	1.19	0.99–1.44
Past smoker	1.16	1.08–1.23	1.11	0.73–1.70
**(Ref.) Non-smoker**	1.00			1.00

^a^(systolic pressure ≥ 140 mmHg or diastolic pressure ≥ 90 mmHg).

**Figure 1:**
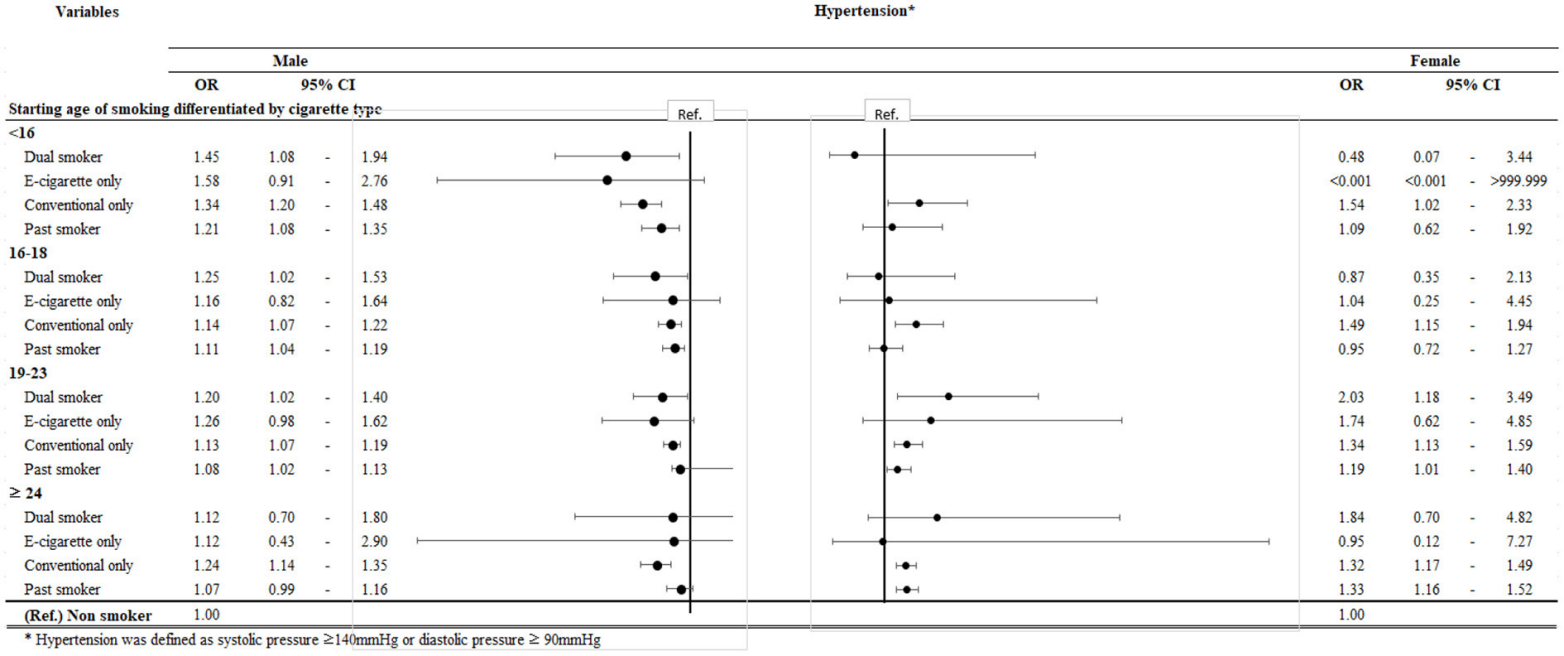
Subgroup analysis by initiation age.

Figure 1 is a forest plot stratified by the age at onset of smoking differentiated by the cigarette type used. A younger age at onset was associated with the tendency of developing hypertension compared to that in non-smokers, especially men [group 1; starting age < 16, OR = 1.45, 95% CI (1.08–1.94); starting age 16–18, OR = 1.25, 95% CI (1.02–1.53); starting age 19–23, OR = 1.20, 95% CI (1.02–1.40); starting age ≥ 24, OR = 1.12, 95% CI (0.70–1.80)]. Similarly, pack-year and age at onset showed a statistically insignificant association in group 2 due to the small sample size because e-cigarettes are relatively novel.

## Discussion

In this cross-sectional study on the types of cigarette use and their association with hypertension, dual smokers (group 1) had the highest odds of developing hypertension [OR = 1.24 95% CI (1.11–1.3.9)] among both men and women [OR = 1.44 95% CI (0.96–2.16)]. This was followed by the e-cigarette only smoking group (group 2); the association was not statistically significant in the conventional only group (group 3) and the past smoker group (group 4).

Previous epidemiological studies have described the effects of dual electronic and conventional cigarette use (22–24). Most people begin using electronic cigarettes after having experienced conventional smoking. Therefore, according to a study about the dual use of cigarettes, approximately 85% of e-cigarette users were dual users, and they had greater nicotine dependence and higher urinary cotinine levels than those of conventional cigarette only smokers (22). It is theorized that the nicotine in tobacco products activates the sympathoadrenal system, thereby increasing the heart rate, BP, and glucose levels (23). This result means that dual smokers tend to have a stronger dependency on nicotine than single use smokers of any type. Moreover, in a study regarding the “high-sensitivity C-reactive protein (hs-CRP),” a marker of inflammation which could act as a predictor of cardiovascular disease, researchers found that dual users had the highest risk of having elevated CRP levels. CRP increase is related to smoking intensity; thus, dual use is not recommended as reported by the study (24). Another study indicated that the reason why e-cigarette only smokers tend to have a decreased risk of disease compared to dual smokers is due to their relatively younger age, lower health risk, and higher socioeconomic status (25). Table 3 supports the proportional relationship between dual and e-cigarette only smokers and a higher possibility of hypertension prevalence as explained above. The highest OR among the types of cigarettes used is congregated in the dual or e-cigarette only group in men. Nevertheless, in women, since their proportion was markedly low in the dual or e-cigarette only smoker group, the highest ORs are dispersed throughout the different types of cigarettes used.

According to previous studies, there are results of the more educated respondents more likely to be aware of e-cigarettes, meaning that they perceive the harmfulness of e-cigarette (26, 27). However, in other studies, those with the higher education level were more likely to be current e-cigarette users (28, 29). In Table 3, our study findings showed that those who have educational level of college or higher and smoke e-cigarette only were most likely to have hypertension in both men and women [men OR: 1.32, 95% CI (1.05–1.65); women OR: 3.34, 95% CI (1.58–7.06)]. Our findings regarding income did not show the relation with e-cigarette smoker, but other studies assume that higher educational level and higher income level is associated with e-cigarette smoking. Moreover, further studies subdividing college or above degrees should be explored.

The biological mechanism underlying the harmful effects of electronic cigarette use on the development of various diseases including hypertension has been previously researched (9, 30–35). Hypertension can lead to various heart diseases, including myocardial infarction (MI) (30–34) and stroke (9, 35). According to a previous study, e-cigarette use is related to endothelial dysfunction (30, 31), oxidative stress (30–32), inflammation (30, 32, 33), and activation of platelet (30, 32) and the sympathetic nervous system (30, 32). This suggests that e-cigarettes could be an independent risk for MI in addition to the baseline risk of smoking (30–33). Another study indicated that nicotine and e-cigarette vaping incite a glucose-deprived environment in the neurovascular unit, resulting in a greater danger from stroke (9, 35). Similar to the previous research results regarding the cardiovascular complications stated above, a recent study reported on the “splenocardiac axis” that functions as an inflammatory signaling network, as the basis of ischemic heart disease. This increase in inflammation could lead to future cardiovascular events among e-cigarette users (34).

In addition to the fact that this is one of the first studies to identify the association between cigarette type practices, specifically e-cigarettes, and hypertension, there are also a few other strengths about the study. There was a recent epidemiologic study on vaping and hypertension (12). Similarly to our study, the previous study by Miller et al. researched different types of cigarettes used, including dual use of conventional and e-cigarettes, or the single use of each. Nevertheless, our study differs from this prior study since our data include only the Korean population. Moreover, to examine the association more clearly, we excluded those who took medication for hypertension. Lastly, we adjusted the association with pack-year or age at onset of smoking (Table 4; Figure 1), given that the amount and duration of smoking are important factors in a study regarding smoking.

This study has certain limitations. Firstly, the proportion of participants who only smoke e-cigarettes was lesser than that of conventional smokers. This caused the CI to be insignificant, even though the ORs were relevant. Since e-cigarette use is novel and people smoking them tend to also smoke conventional cigarettes, the numbers were uneven. On account of e-cigarette use being novel, young people tend to use it more, and single smokers in smoking studies are mostly conventional smokers; this group is usually compared with dual smokers (10, 36, 37). Second, although variables of pack-year and age at onset of conventional smoking were adjusted, some level of residual confounding was likely to exist among current exclusive e-cigarette smokers due to varying levels of lifetime exposure to e-cigarette smoking. The data from KDCA does not include information on the amount and duration of e-cigarette smoking. Moreover, family history related to hypertension or other chronic diseases should be adjusted for further studies when key variable is a type of chronic disease, which is highly affected by genetic factors. Third, since this was a survey on the use of cigarettes, it is possible that the female group did not answer the questions honestly. In Korea, due to cultural awareness, women tend to hide their smoking behavior (38). Therefore, the female group values may be less reliable. Lastly, further studies regarding e-cigarette only smokers using panel study designs other than cross-sectional studies, such as longitudinal analysis, are needed.

In conclusion, we found that among smokers of the two cigarette types, people who only smoke e-cigarettes and dual smokers of e- and conventional cigarettes are most likely to have a high prevalence of hypertension (SBP ≥ 140 mmHg or DBP ≥ 90 mmHg). Further research on the adverse effects of e-cigarette use is warranted. Moreover, e-cigarettes should be used after careful verification of the liquid components.

## Data availability statement

Data used in the study were accessed from the KCHS implemented in 2019 by the Korea Center for Disease Control and Prevention Agency (KDCA), and further inquiries can be directed to the website (https://chs.kdca.go.kr/chs/rdr/rdrInfoPledgeMain.do).

## Ethics statement

The KCHS received the Korea Centers for Disease Control and Prevention(KCDC) IRB approval (2016-10-01-P-A) in 2016. From 2017, the ethics approval for the KCHS was waived by the KCDC IRB as it does not fall under human subject research based on the enforcement rule of the bioethics and safety act. The patients/participants provided their written informed consent to participate in this study.

## Author contributions

SK and JS contributed to the conception, design, acquisition, analysis and interpretation of the data, and drafted the manuscript. SK and SJ contributed to the conception and design of the study. HJ and JK worked on analyzing the data as designed. HJ and MP participated in the interpretation of the data. SK and JL wrote the manuscript. E-CP and JS critically revised the manuscript. All authors gave final approval and agree to be accountable for all aspects of the work ensuring integrity and accuracy.

## Conflict of interest

The authors declare that the research was conducted in the absence of any commercial or financial relationships that could be construed as a potential conflict of interest.

## Publisher's note

All claims expressed in this article are solely those of the authors and do not necessarily represent those of their affiliated organizations, or those of the publisher, the editors and the reviewers. Any product that may be evaluated in this article, or claim that may be made by its manufacturer, is not guaranteed or endorsed by the publisher.
